# Combining Haar Wavelet and Karhunen Loeve Transforms for Medical Images Watermarking

**DOI:** 10.1155/2014/313078

**Published:** 2014-04-07

**Authors:** Mohamed Ali Hajjaji, El-Bay Bourennane, Abdessalem Ben Abdelali, Abdellatif Mtibaa

**Affiliations:** ^1^LE2I Laboratory, Burgundy University, Dijon, France; ^2^Electronics and Microelectronics Laboratory, University of Monastir, Tunisia

## Abstract

This paper presents a novel watermarking method, applied to the medical imaging domain, used to embed the patient's data into the corresponding image or set of images used for the diagnosis. The main objective behind the proposed technique is to perform the watermarking of the medical images in such a way that the three main attributes of the hidden information (i.e., imperceptibility, robustness, and integration rate) can be jointly ameliorated as much as possible. These attributes determine the effectiveness of the watermark, resistance to external attacks, and increase the integration rate. In order to improve the robustness, a combination of the characteristics of Discrete Wavelet and Karhunen Loeve Transforms is proposed. The Karhunen Loeve Transform is applied on the subblocks (sized 8 × 8) of the different wavelet coefficients (in the HL2, LH2, and HH2 subbands). In this manner, the watermark will be adapted according to the energy values of each of the Karhunen Loeve components, with the aim of ensuring a better watermark extraction under various types of attacks. For the correct identification of inserted data, the use of an Errors Correcting Code (ECC) mechanism is required for the check and, if possible, the correction of errors introduced into the inserted data. Concerning the enhancement of the imperceptibility factor, the main goal is to determine the optimal value of the visibility factor, which depends on several parameters of the DWT and the KLT transforms. As a first step, a Fuzzy Inference System (FIS) has been set up and then applied to determine an initial visibility factor value. Several features extracted from the Cooccurrence matrix are used as an input to the FIS and used to determine an initial visibility factor for each block; these values are subsequently reweighted in function of the eigenvalues extracted from each subblock. Regarding the integration rate, the previous works insert one bit per coefficient. In our proposal, the integration of the data to be hidden is 3 bits per coefficient so that we increase the integration rate by a factor of magnitude 3.

## 1. Introduction

Medical imaging is an important tool and essential in the diagnosis and decisions made by health professionals. In this context, several techniques and imaging models have been proposed by many authors. Among these techniques, the most common are MRI, Echographic images, Radiographic images, and Mammographic images. These techniques have proven to be very successful in diagnosing, and so forth.

However, these diagnoses are often insufficient or inconclusive due to the complexity of the diseases or the limitation of the imaging techniques themselves. Therefore, developing new tools enable physicians, usually located in different regions or countries, to collaborate remotely, in order to get a better diagnosis which has become increasingly widespread and necessary. This trend is known as telemedicine. The main problem of these endeavors arises when managing the integrity and confidentiality of data on the internet against pirates. Several solutions based on the use of access control techniques exist, but they remain elusive and hence the appearance of watermarking techniques in order to ameliorate the security control of the network in which those medical images [1–3] are shared [4].

In the general case, digital watermarking is a technique that consists in hiding information in a digital document (indelible or invisible depending in the nature of the document and hence the name) to ensure security in the intended service (copyright, integrity, and nonrepudiation). A special feature of watermarking compared to other techniques is that the watermark is intimately linked to the associated document and resistant to attacks. Therefore, the watermarking is theoretically independent of the file format and it can be detected or extracted even if the document has changed or is incomplete.

This paper is organized as follows.  Section 2 presents the problem and criteria for a digital watermarking system. Related work is provided in Section 3.  Section 4 deals with the proposed watermarking technique.  Section 5 presents a methodology for adjusting of the visibility factor. The insertion and extraction of the watermark are, respectively, represented in the Sections 6 and 7. The simulation, validation, and the evaluation of the proposed algorithm are represented in the Section 8. Finally, Section 9 concludes the paper.

## 2. Problem and Criteria for a Digital Watermarking System

Watermarking techniques require various features according to their areas of application and their underlying objectives. The hidden watermark in an image must satisfy three basic conditions: robustness, imperceptibility, and capacity. They are not independent of each other; rather they are closely related [5].

### 2.1. Imperceptibility

The watermark should not affect the quality of the original image after any watermarking operation. Cox et al. [6] define the imperceptibility as visual similarity between the original and watermarked images. The watermark must be inserted in a way that is completely invisible to the Human Visual System (HVS) [7].

The insertion process must not damage the host image; that is, the watermarked image has to be visually equivalent to the original image. Not only the image should not be distorted, but also the watermark must be hidden and invisible, otherwise it could be easily removed. In general, the more the imperceptibility is low, the more the robustness and capacity are strong.

### 2.2. Capacity

The ability of a watermarking system refers to the ratio of the “Amount of data” to hide on the “size of the host document” [8]. Sometimes the size of the watermark is limited just to 1 bit. In general, the more the capacity is low, the more the robustness and the imperceptibility are strong.

### 2.3. Robustness

Robustness [9] is the resistance of the watermark system against intentional transformations of the watermarked image. These transformations can be of geometric type (rotation, cropping); they can also include all types of degradation of the image frequencies (lossy compression, high pass filter, or low pass filter). In general, the more the robustness is low, the more the capacity and the imperceptibility are strong.

**Figure 1:**
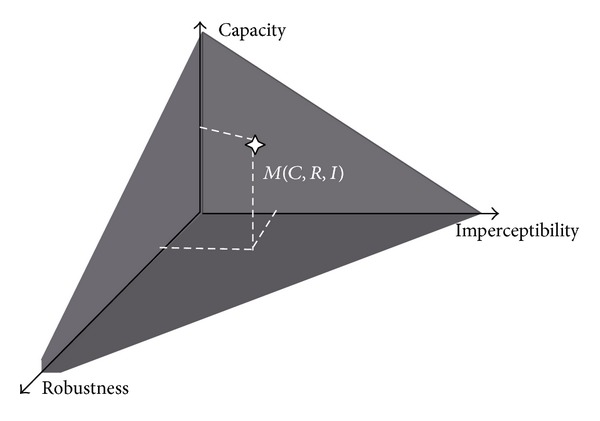
Pyramidal compromise.

Many ongoing efforts on watermarking techniques are being carried out with the aim of optimizing these three aforementioned criteria. Moreover, these three parameters are closely related to a pattern of watermarking images so any modification of one of these factors influences directly the others (Figure 1).

## 3. Related Works

In this section we present an in-depth review of digital image watermarking techniques. It describes the previous works which had been done on digital watermarking by using DWT technique.

In [10] Anuradha and Singh proposed a watermarking system aimed at protecting the copyright and the control of the integrity related to the digital products. The multiresolution space, obtained by Haar Wavelet Transform, is used for hiding the watermark in the 3rd level of decomposition. The horizontal, vertical, and diagonal subbands are processed for inserting the totality of the watermark.

In [11] Kashyap proposed a blind watermarking system in the 3rd level Discrete Wavelet Transform. The idea consists in hiding a watermark in the low frequency subband. For insertion, Kashyap defined the visibility factor in function of scaling factor of the subbands of the original image and the watermark. The insertion step follows the next formula:

(1) $$\begin{array}{c} \text{WMI}=K\times \left(\text{L}{\text{L}}_{3}\right)+q\times \left(\text{W}{\text{M}}_{3}\right), \end{array}$$

where WMI is low frequency component of watermarked image, LL_3_ is the low frequency component of the original image obtained by 3-level DWT, and WM_3_ is the low frequency component of Watermark image. *K* and *q* are the scaling factors of the original image and the watermark, respectively.

In [12] Peter Cika describes a new watermarking method based on two-dimensional wavelet transform and the Singular-Value Decomposition. The diagonal matrix (after decomposition on SVD) *S* is used for inserting the watermark after decomposition on wavelet transform.

In [13] Kannammal and Subha Rani proposed a watermarking system for medical images authentication. The proposed space, for hiding information, is the low frequency subband after the first level of the wavelet transform decomposition. The components chosen to hide the watermark are selected by the *N* random numbers generated, which have the integers value from 0 to *K*. The watermark is the hash value of the MSBs of different coefficients selected to hide the watermark. The output of the hash function is embedded into the selected coefficients, and it is combined with the MSBs to get the watermarked coefficients.

In [14] Rawat and Raman proposed a dual watermarking scheme based on Discrete Wavelet Transform (DWT), Wavelet Packet Transform (WPT) with best tree- and Singular-Value Decomposition (SVD). Using subsampling of the cover image, four subimages are obtained and they calculate their SVD values. They chose *X* and *Y* two subimages which contain the highest sum of SVD for embedding two different watermarks. The watermarks W1 and W2 are embedded with two different methods. For embedding the first watermark, they apply the WPT in l level; afterwards the best tree is chosen. The insertion step consists in combining the two diagonals matrices of the SVD transforms of the watermark and the best tree. For the second watermark, the DWT in l level is applied. Then they calculate the SVD of different subbands. For the insertion step, they applied the same method for embedding W1.

In [15] Yang et al. presented a watermarking algorithm based on the Integer Wavelet Transform (IWT). The low-high (LH) and high-low (HL) subbands are used for hiding the watermark. The simulations show good results in terms of the watermarked image quality. The proposed schema is robust against several attacks such as brightness, inversion, and compression attack.

In [16] Makbol and Khoo presented a watermarking algorithm based on the Redundant Discrete Wavelet Transform (RDWT) and the Singular-Value Decomposition. The watermark to be inserted is a gray scale image. The insertion step is applied directly in the SVD components of the RDWT subbands of the host image.

In [17] Latif proposed a new watermarking method based on the Parametric Slant-Hadamard Transform. To ameliorate the imperceptibility factor, the authors have exploited the masking characteristics of the Human visual System using Fuzzy Inference System. The experimental results show that the proposed technique has a high imperceptibility as well as a high robustness against several attacks.

For controlling the authentication, Al-Saif et al. [18] propose a new watermarking method applied on the gray scale image. The proposed space is the Karhunen Loeve Transform. The eigenvalues are used for hiding the watermark.

Most methods found in the literature fail to optimize the compromise existing in the digital watermarking (robustness, imperceptibility, and capacity). Moreover, all previous works use a logo to be hidden in the original image which is not the case concerning medical imaging.

In this paper, we propose a new simple methodology applied on medical imaging. The basic idea consists in preserving the visual quality of the original image to increase the rate related to data to be inserted and to enhance the robustness of our method against many attacks.

## 4. Proposed Watermarking Technique

In this section, we explain the proposed algorithm for embedding the totality of the patient's data in a medical image. Before the insertion, the patient's data undergoes many steps with the aim at increasing the level of integration and better ensuring its extraction after applying different attacks. The insertion procedure is done by adding to the image frequency components, proper to the original image, data related to the patient.

The following expression explains the integration procedure of the watermark on the image frequency values:

(2) $$\begin{array}{c} Y^{\prime }\left(i\right)=Y\left(i\right)+\alpha \times W\left(i\right), \end{array}$$

with

- *Y*(*i*) being the *i*th old coefficient to support the watermark;
- *Y*′(*i*) being the *i*th new watermarked coefficient;
- *W*(*i*) being the *i*th bit to hide;
- *α* being named often the visibility factor. The visibility factor *α* is an important factor in the watermarking system. If *α* is big, we win in terms of the robustness but we lose in terms of imperceptibility and vice versa.

In order to achieve an optimal imperceptibility, it is necessary to go through a stage of preparation of the insertion space. The rationale behind our contribution is based on the use of the subbands obtained after applying the second level wavelet decomposition then followed by the subdivision stage in which they are further subdivided into smaller subblocks sized 8 × 8. After that, we calculate the Karhunen Loeve Transform on each subblock and embed the watermark. Finally, the inverse transforms are performed to obtain watermarked image.

**Figure 2:**
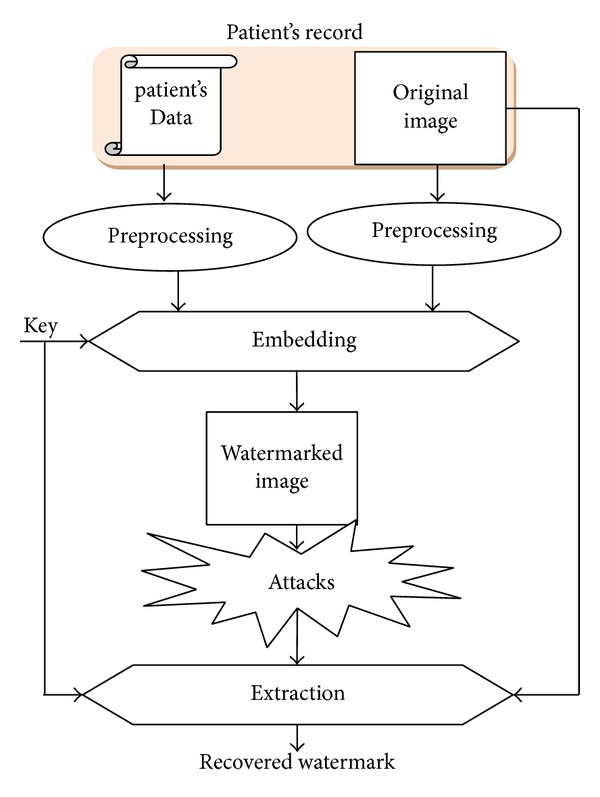
General digital watermarking algorithm.

To evaluate the performance of our algorithm; an extraction phase is applied on the watermarked image. In addition, the Normalized Cross-Correlation (NC) is carried out to evaluate the similarity between the original and the extracted data. The proposed process is summarized in Figure 2.

### 4.1. Determining the Regions to Be Watermarked

In our method, we propose to use a combination of two transformations on the original image for embedding the watermark.

First, the second level Haar Wavelet Transform [19] is carried out. In this stage, each subband is formed of *N* subblocks of 8 × 8 coefficients. Second, we chose the frequency subbands, such as HH2, HL2, and LH2. The principal characteristic of the high frequency subbands is that the edges and textures usually are found here more prominently. They are used with the aim to accord the visibility factor with psychovisual characteristics of these subbands. Third, we subdivide HH2, HL2, and LH2 into subblocks sized 8 × 8. Finally, the Karhunen Loeve Transform is applied.

**Figure 3:**
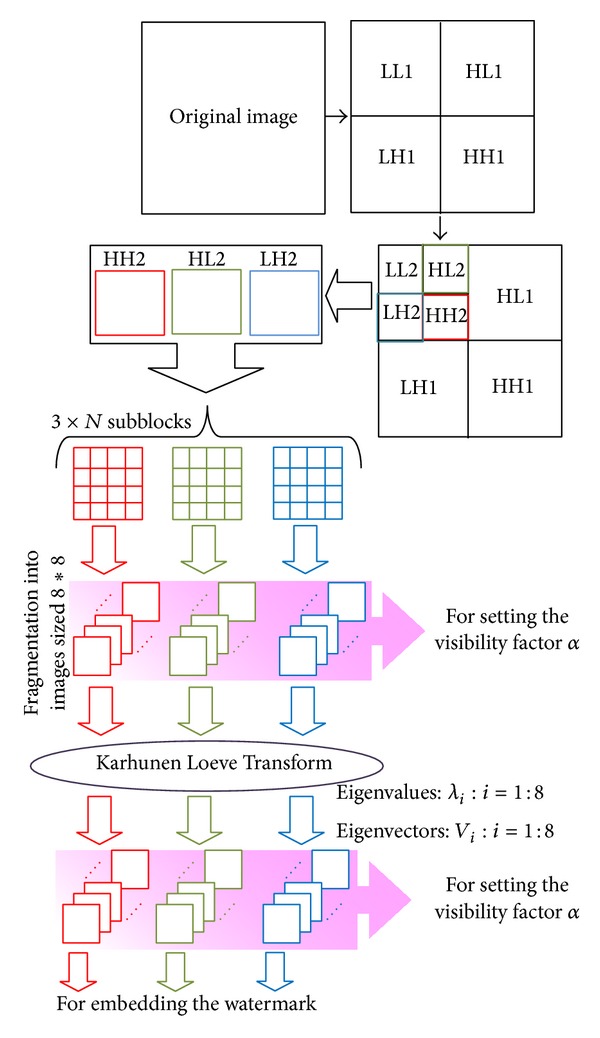
Preparation of insertion plans.

Figure 3 presents the steps for the preparation of the regions where the watermark will be hidden.

### 4.2. Preparation of the Watermark

Most of the algorithms, found in the literature for embedding watermarks, propose to integrate directly binary data in frequency components chosen in the original image, after multiplication by a visibility factor. However, it is necessary in the medical imaging domain to take into account the integration rate given that the amount of information to be hidden in the host image (name, age, sex, diagnosis, and signature) can be significant. The idea consists in using the data from the patient (the watermark) as an index for insertion, after its transformation into an octal sequence. This index services as an address to access a row among 8 of each sub-block.

Algorithm 1 illustrates the typical example of the data from a patient and which can be inserted in the image.

The computation steps for the index table are performed as follows.

- For controlling the authenticity of the patient's record, a signature owned by the hospital center is generated. For this purpose, the SHA-1 hash function [20] is used.
- Convert the rest of data file into a binary sequence and concatenate it with the binary signature. Thus, we obtain a message which will be coded in the following steps.
- For improving the reliability and the detection of the hidden data, we apply the concept of channel coding. So, the message is coded by the serial Turbocode [21].
- The whole of coded binary sequence is assumed to be equal to “3 × *P*,” where *P* is less or equal to 3 × *N*. 3 × *N* is the total number of subblocks in subbands (LH2, HL2, and HH2). The binary sequence is then converted into the octal representation format. We then generate three addressing tables of identical sizes equal to *N*. We start by filling the first table to contain addresses for indexing LH2 subband by the *N* first values of the octal presentation sequence. By the remaining octal indexing values, we fill the second table related to HL2 and the third table related to HH2, respectively.
- For the insertion process, we start by the *N* subblocks of 8 × 8 values each; extracted from HL2, the *N* first values of the first indexing table are selected for their uses during the insertion in this subband. Afterwards, the same process is applied on the *N* second values of the indexing table related to LH2. The rest of indexing table data is then used for the insertion in the third subband HH2.

**Figure 4:**
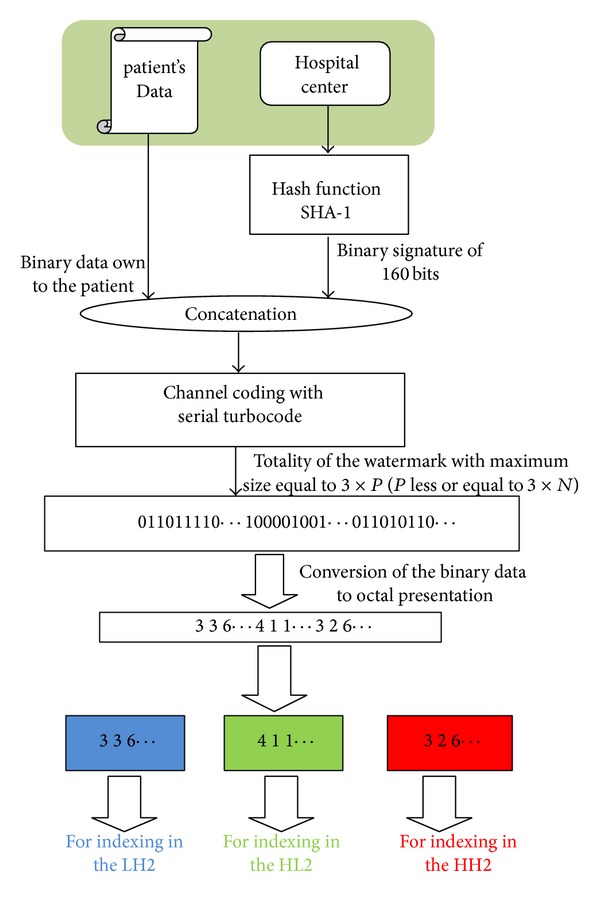
Preparation of the indexing tables.

Figure 4 summarizes the different steps to follow to prepare the index table related to the original data to be inserted. These tables are subsequently used for indexing a pseudorandom sequence generated with a secret key for embedding and extracting of the watermark.

## 5. Adjustment of the Visibility Factor

The principal problem in the watermarking domain is the compromise between imperceptibility, robustness, and the integration rate. However, the problem of the integration rate can be solved by collecting the different data to be inserted, with the goal of minimizing the necessary modifications on the image. Concerning the robustness and the imperceptibility, they are directly related to the visibility factor known as “*α*.” Therefore, it is very important to take into account this compromise. A good compromised can be achieved by weighting the visibility factor according to the Human Visual System HVS [7]. One of the main contributions of this work consists in proposing a method in which data is inserted in the LH2, HL2, and HH2 subbands after their decompositions into Karhunen Loeve components [22]. The idea is to weight the value of alpha according to each subblock.

After the multiresolution transform, the totalities of the subbands (HL2, LH2, and HH2) are divided into subblocks sized 8 × 8.

For the adjusting of the visibilities factors values, a two-step algorithm is applied. In the first step, the visibility factors are defined in function of contrast sensibility, entropy sensibility, and homogeneity factor, which are extracted from the subblocks of the different subbands (LH2, HL2, and HH2). Proportionally to the different subbands, these visibility factors are named, respectively, *α*_*LH*⁡2_, *α*_HL2_, and *α*_HH2_.

In the next step, the sum of *α*_*LH*⁡2_, *α*_HL2_, and *α*_HH2_ is used to determine the final visibility factor named *α*_*LH*⁡2_Final_, *α*_HL2_Final_, and *α*_HH2_Final_. Their weights are based on the properties of the Karhunen Loeve space.

**Figure 5:**
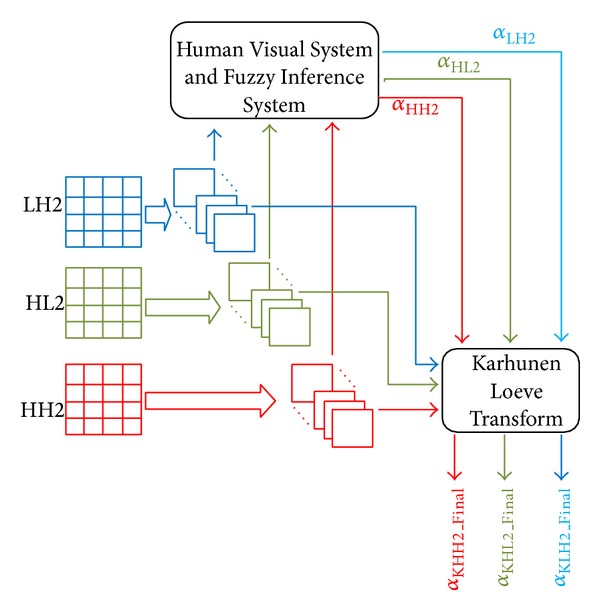
Overview of determination of the visibility factor.

Figure 5 describes the general steps to follow for setting the visibilities factors values.

The remainder of this section details how the visibilities factors are obtained, depending on LH2, HL2, and HH2 and on the eigenvalues extracted using the Karhunen Loeve Transform.

### 5.1. Fuzzy Inference System and Frequency Subbands for Determination of the Visibility Factor

**Figure 6:**
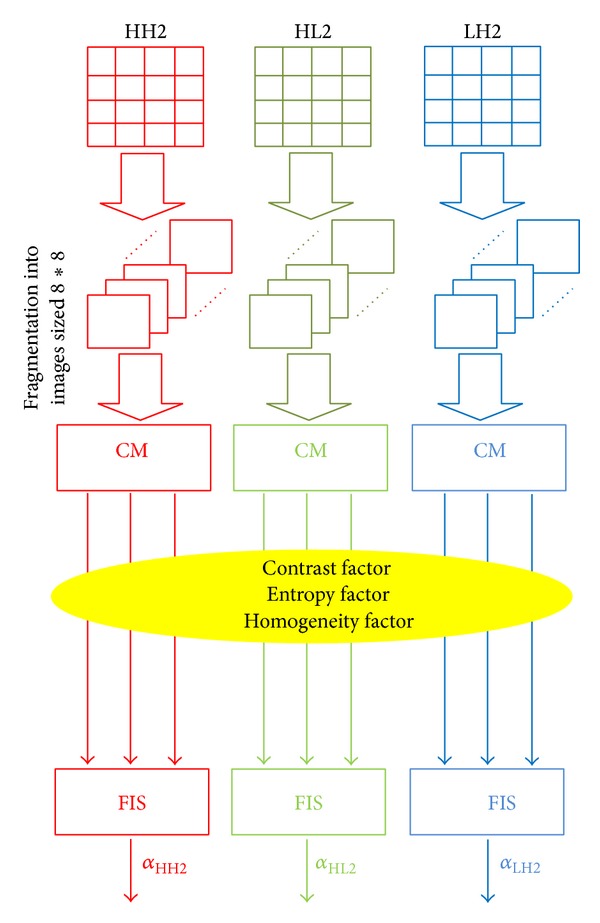
The steps for determining the primary visibility factor for each subband.

Figure 6 summarizes the procedure for determining the primary values of the optimal visibility factors related to the visual characteristics of the different subbands.

In the HVS, among the most important factors, three properties are employed in the watermarking algorithm: contrast sensitivity, entropy sensitivity, and homogeneity sensitivity. These factors are extracted by Cooccurrence matrix (CM) [23].

The entropy sensitivity measures the degree of disorder on the image. It attains high values for a random texture; thus, the more the entropy is high, the more the reinforcement of the visibility factor is possible.

The contrast sensitivity is often important when moving from a low gray level (or high) to a high gray level (or low). This is the case in the transition regions in an image. In this case, the more the contrast sensitivity is high, the more the reinforcement of *α* is possible.

The homogeneity sensitivity reflects the fact that we often encounter or not the same pair of pixels separated by the translation “*t*” (after application of the cooccurrence matrix). Its value is high if the image is a gray uniform. Therefore, when the homogeneity sensitivity is small, it is possible to reinforce the visibility factor *α*. In the proposed method, we integrate an intelligent process which is able to automate the value of the visibility factor in terms of the psychovisual quality of the corresponding insertion space.

This process is used in the so-called Fuzzy Inference Systems: in such a system, the contrast sensitivity *C*_*k*_, homogeneity sensitivity *H*_*k*_, and the entropy sensitivity *E*_*k*_ are taken as inputs for estimating the adaptive weight visibility factors *α*_*k*_.

Using the Fuzzy Inference System enables us to increase the visibility factor *α* in the less sensitivity areas (High texture, important contrast, and small homogeneity), while at the same time decreasing the value of the visibility factor in more sensitive areas (important homogeneity, small contrast, and small entropy). For this purpose, the fuzzy logic [24] is used.

In general, it is based on the idea of the human experts, by their subjective and qualitative descriptions of behavior of watermarking method with natural language. The principle of the fuzzy logic is similar to the human behavior. It is based on the linguistic variables related to the human language. Moreover these variables are determined by some empirically experiments.

**Figure 7:**
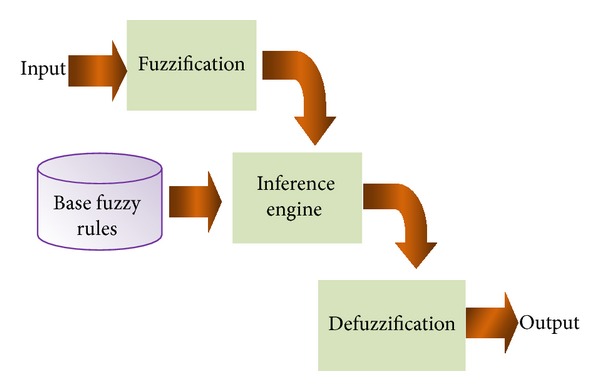
A Fuzzy Inference System.

As shown in Figure 7, a Fuzzy Inference System is composed of three principal blocks:

- a Fuzzification step converts the numeric values to degrees of membership of different fuzzy set;
- an Inference engine step that contains different rules;
- Defuzzification step to generate a net worth for *α* which represents the output of the fuzzy system.

When applying fuzzy logic to image watermarking, it is very important to determine the essential elements to find the optimal value for adjusting *α*. Those elements are the fuzzy variables, inference rules, and the membership functions.

**Figure 8:**
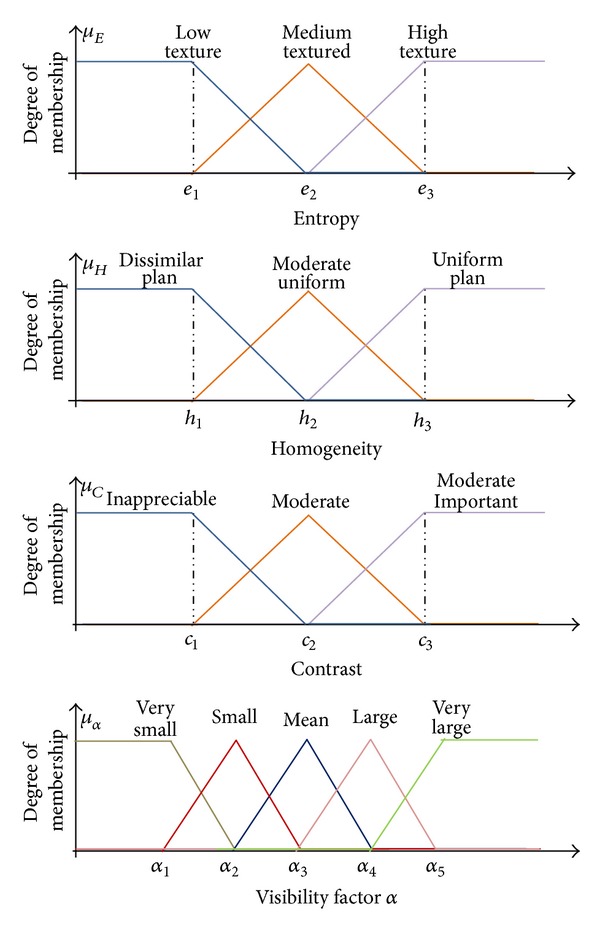
Dynamic Membership Functions and mapping of their input/output variables to fuzzy sets.

The Fuzzy variables, also called linguistic variables, do not only take binary values but also have an infinite number of possible values between the “logic true” and the “logic false.” The Fuzzy variables are involved in the description of certain situations, phenomenon, or process generally containing fuzzy qualifiers. For example, (as shown in Figure 8) for the entropy sensitivity we use the following fuzzy variables: Low texture, Medium textured, and High texture.

Inference rules and fuzzy rules are used for linking the different variables of the fuzzy system with its input variables and fuzzy outputs. These rules come in the following form:

 If (condition 1) and/or condition (*X*) then (action on the outputs).

We summarize that these rules make the experience of the expert and they are usually not uniquely definable as each individual creates his own rules. To do this, we define two notions:

- the membership functions that define the degree of truth of fuzzy variable depending on the input;
- the fuzzy intervals which determine the number of fuzzy variables.

The input and output membership functions exploited are shown in Figure 8. It must be noted that this approach enables us to adjust the entropy (or homogeneity and the contrast) membership function in such a manner that best fits to the properties of the image.

In consequence, the approximations of the inferred values are optimized and used to generate an adaptive value strength for the inserted watermark. The membership functions used in our algorithm are the triangular and trapezoidal functions.

Concerning the evaluation of the output system, in fuzzy logic, the Defuzzification phase is used for translating the fuzzy values into numerical values. This step is done by using the membership functions. In our approach, the inference results are subsequently computed by means of the minimum-maximum Defuzzification method. In this manner, we determine the initial visibility factors noted *α*_*LH*⁡2_, *α*_HL2_, and *α*_HH2_.

### 5.2. Impact of the Karhunen Loeve Transform on the Visibility Factor

Given an image *I* (in our case sized 8 × 8) formed by 8 columns of 8 rows each. Let us call those columns *I*_*i*_ for *i* = {1,2,…, 8}; we calculate the covariance matrix of the image *C*_*I*_ [22]:

(3) $$\begin{array}{c} C_{I}=E\left\{\left(I-m_{I}\right){\left(I-m_{I}\right)}^{T}\right\},\\ T\;\text{indicates}\;\text{the}\;\text{matrix}\;\text{transpose}. \end{array}$$

Since *C*_*I*_ is real and symmetric, it is always possible to find a set of 8 orthonormal eigenvectors. Let *v*_*i*_ and *λ*_*i*_, *i* = {1,2,…, 8}, be the eigenvectors and corresponding eigenvalues of *C*_*I*_, arranged in decreasing order so that *λ*_*i*_ ≥ *λ*_*i*+1_ for *i* = {1,2,…, 8}. Let *V* be a matrix whose rows are formed of the eigenvectors of *C*_*I*_, arranged so that the first row of *V* is the eigenvector corresponding to the largest eigenvalue, and the last row is the eigenvector corresponding to the smallest eigenvalue. The Karhunen Loeve Transform (also known as the Principal Components Transform) and its inverse (IKLT) may then be defined as

(4) KLT=V×I,IKLT=VT×KLT,with  V:eigenvector  matrix  extracted  from  IVT:eigenvector  transpose  matrix.

This operation has a few key features. First, the Karhunen Loeve Transform decorrelates the signal components of KLT, suggesting that we could reconstruct each Karhunen Loeve component separately in the Karhunen Loeve domain as a sequence of independent reconstructions. Second, the Karhunen Loeve Transform tends to compact the original block content with the eigenvectors stemming of the eigenvalues. This advantage will be used for the weighting again of the visibility factors (already defined in terms of multiresolution space).

At the Karhunen Loeve Transform, different eigenvectors *V*_*i*_, for *i* = {1,2,…, 8}, present the main directions that carry energy in the image.

However, the eigenvectors are closely related to the eigenvalues our idea is to weight the value of alpha in function to different eigenvalues *λ*_*i*_.

Whether *λ*_*i*_ ≥ *λ*_*i*+1_ for *i* = {1,2,…, 8}, in this case the value of *α* is inversely proportional to *λ* values; that is, *α*_*i*_ ≤ *α*_*i*+1_ (*i* = {1,2,…, 8}). In this case if, for example, the insertion will take place on *V*_5_, the value of *α* is equal to *α*_5_.

In conclusion, after determining *α* by the psychovisual characteristics, it will be reweighted (depending on the column *V*_*i*_ just before the calculation of the Karhunen Loeve transform matrix) for forming the final value of the visibility factor *α*.

## 6. Proposed Insertion Algorithm

### 6.1. Choice of Components to Support the Watermark

Suppose that the input subblock, sized 8 × 8, is represented by a matrix *I*. The Karhunen Loeve Transform can be represented by

(5) $$\begin{array}{c} \text{KLT}=V\times I,\\ \left[\begin{array}{cccccccc} \text{KL}{\text{T}}_{1} & \text{KL}{\text{T}}_{9} & \text{KL}{\text{T}}_{17} & \text{KL}{\text{T}}_{25} & \text{KL}{\text{T}}_{33} & \text{KL}{\text{T}}_{41} & \text{KL}{\text{T}}_{49} & \text{KL}{\text{T}}_{57}\\ \text{KL}{\text{T}}_{2} & \text{KL}{\text{T}}_{10} & \text{KL}{\text{T}}_{18} & \text{KL}{\text{T}}_{26} & \text{KL}{\text{T}}_{34} & \text{KL}{\text{T}}_{42} & \text{KL}{\text{T}}_{50} & \text{KL}{\text{T}}_{58}\\ \text{KL}{\text{T}}_{3} & \text{KL}{\text{T}}_{11} & \text{KL}{\text{T}}_{19} & \text{KL}{\text{T}}_{27} & \text{KL}{\text{T}}_{35} & \text{KL}{\text{T}}_{43} & \text{KL}{\text{T}}_{51} & \text{KL}{\text{T}}_{59}\\ \text{KL}{\text{T}}_{4} & \text{KL}{\text{T}}_{12} & \text{KL}{\text{T}}_{20} & \text{KL}{\text{T}}_{28} & \text{KL}{\text{T}}_{36} & \text{KL}{\text{T}}_{44} & \text{KL}{\text{T}}_{52} & \text{KL}{\text{T}}_{60}\\ \text{KL}{\text{T}}_{5} & \text{KL}{\text{T}}_{13} & \text{KL}{\text{T}}_{21} & \text{KL}{\text{T}}_{29} & \text{KL}{\text{T}}_{37} & \text{KL}{\text{T}}_{45} & \text{KL}{\text{T}}_{53} & \text{KL}{\text{T}}_{61}\\ \text{KL}{\text{T}}_{6} & \text{KL}{\text{T}}_{14} & \text{KL}{\text{T}}_{22} & \text{KL}{\text{T}}_{30} & \text{KL}{\text{T}}_{38} & \text{KL}{\text{T}}_{46} & \text{KL}{\text{T}}_{54} & \text{KL}{\text{T}}_{62}\\ \text{KL}{\text{T}}_{7} & \text{KL}{\text{T}}_{15} & \text{KL}{\text{T}}_{23} & \text{KL}{\text{T}}_{31} & \text{KL}{\text{T}}_{39} & \text{KL}{\text{T}}_{47} & \text{KL}{\text{T}}_{55} & \text{KL}{\text{T}}_{63}\\ \text{KL}{\text{T}}_{8} & \text{KL}{\text{T}}_{16} & \text{KL}{\text{T}}_{24} & \text{KL}{\text{T}}_{32} & \text{KL}{\text{T}}_{40} & \text{KL}{\text{T}}_{48} & \text{KL}{\text{T}}_{56} & \text{KL}{\text{T}}_{64} \end{array}\right]\\ =\left[\begin{array}{cccccccc} v_{1} & v_{9} & v_{17} & v_{25} & v_{33} & v_{41} & v_{49} & v_{57}\\ v_{2} & v_{10} & v_{18} & v_{26} & v_{34} & v_{42} & v_{50} & v_{58}\\ v_{3} & v_{11} & v_{19} & v_{27} & v_{35} & v_{43} & v_{51} & v_{59}\\ v_{4} & v_{12} & v_{20} & v_{28} & v_{36} & v_{44} & v_{52} & v_{60}\\ v_{5} & v_{13} & v_{21} & v_{29} & v_{37} & v_{45} & v_{53} & v_{61}\\ v_{6} & v_{14} & v_{22} & v_{30} & v_{38} & v_{46} & v_{54} & v_{62}\\ v_{7} & v_{15} & v_{23} & v_{31} & v_{39} & v_{47} & v_{55} & v_{63}\\ v_{8} & v_{16} & v_{24} & v_{32} & v_{40} & v_{48} & v_{56} & v_{64} \end{array}\right]\times \left[\begin{array}{cccccccc} I_{1} & I_{2} & I_{3} & I_{4} & I_{5} & I_{6} & I_{7} & I_{8}\\ I_{9} & I_{10} & I_{11} & I_{12} & I_{13} & I_{14} & I_{15} & I_{16}\\ I_{17} & I_{18} & I_{19} & I_{20} & I_{21} & I_{22} & I_{23} & I_{24}\\ I_{25} & I_{26} & I_{27} & I_{28} & I_{29} & I_{30} & I_{31} & I_{32}\\ I_{33} & I_{34} & I_{35} & I_{36} & I_{37} & I_{38} & I_{39} & I_{40}\\ I_{41} & I_{42} & I_{43} & I_{44} & I_{45} & I_{46} & I_{47} & I_{48}\\ I_{49} & I_{50} & I_{51} & I_{52} & I_{53} & I_{54} & I_{55} & I_{56}\\ I_{57} & I_{58} & I_{59} & I_{60} & I_{61} & I_{62} & I_{63} & I_{64} \end{array}\right]. \end{array}$$

After performing the matrix multiplication for *V* × *I*, the 64 components KLT_1_, KLT_2_,…, KLT_63_, and KLT_64_ are given by

(6) KLT1=v1×I1+v9×I9+v17×I17+v25×I25+v33×I33+v41×I41+v49×I49+v57×I57,KLT2=v2×I2+v10×I10+v18×I18+v26×I25+v34×I34+v42×I42+v50×I50+v58×I58,⋮KLT63=v7×I7+v15×I15+v23×I23+v31×I31+v39×I39+v47×I47+v55×I55+v63×I63,KLT64=v8×I8+v16×I16+v24×I24+v32×I32+v40×I40+v48×I48+v56×I56+v64×I64.

The Inverse Karhunen Loeve Transform can be represented as

(7) $$\begin{array}{c} \text{IKLT}=V^{T}\times \text{KLT},\\ \left[\begin{array}{cccccccc} \text{IKL}{\text{T}}_{1} & \text{IKL}{\text{T}}_{9} & \text{IKL}{\text{T}}_{17} & \text{IKL}{\text{T}}_{25} & \text{IKL}{\text{T}}_{33} & \text{IKL}{\text{T}}_{41} & \text{IKL}{\text{T}}_{49} & \text{IKL}{\text{T}}_{57}\\ \text{IKL}{\text{T}}_{2} & \text{IKL}{\text{T}}_{10} & \text{IKL}{\text{T}}_{18} & \text{IKL}{\text{T}}_{26} & \text{IKL}{\text{T}}_{34} & \text{IKL}{\text{T}}_{42} & \text{IKL}{\text{T}}_{50} & \text{IKL}{\text{T}}_{58}\\ \text{IKL}{\text{T}}_{3} & \text{IKL}{\text{T}}_{11} & \text{IKL}{\text{T}}_{19} & \text{IKL}{\text{T}}_{27} & \text{IKL}{\text{T}}_{35} & \text{IKL}{\text{T}}_{43} & \text{IKL}{\text{T}}_{51} & \text{IKL}{\text{T}}_{59}\\ \text{IKL}{\text{T}}_{4} & \text{IKL}{\text{T}}_{12} & \text{IKL}{\text{T}}_{20} & \text{IKL}{\text{T}}_{28} & \text{IKL}{\text{T}}_{36} & \text{IKL}{\text{T}}_{44} & \text{IKL}{\text{T}}_{52} & \text{IKL}{\text{T}}_{60}\\ \text{IKL}{\text{T}}_{5} & \text{IKL}{\text{T}}_{13} & \text{IKL}{\text{T}}_{21} & \text{IKL}{\text{T}}_{29} & \text{IKL}{\text{T}}_{37} & \text{IKL}{\text{T}}_{45} & \text{IKL}{\text{T}}_{53} & \text{IKL}{\text{T}}_{61}\\ \text{IKL}{\text{T}}_{6} & \text{IKL}{\text{T}}_{14} & \text{IKL}{\text{T}}_{22} & \text{IKL}{\text{T}}_{30} & \text{IKL}{\text{T}}_{38} & \text{IKL}{\text{T}}_{46} & \text{IKL}{\text{T}}_{54} & \text{IKL}{\text{T}}_{62}\\ \text{IKL}{\text{T}}_{7} & \text{IKL}{\text{T}}_{15} & \text{IKL}{\text{T}}_{23} & \text{IKL}{\text{T}}_{31} & \text{IKL}{\text{T}}_{39} & \text{IKL}{\text{T}}_{47} & \text{IKL}{\text{T}}_{55} & \text{IKL}{\text{T}}_{63}\\ \text{IKL}{\text{T}}_{8} & \text{IKL}{\text{T}}_{16} & \text{IKL}{\text{T}}_{24} & \text{IKL}{\text{T}}_{32} & \text{IKL}{\text{T}}_{40} & \text{IKL}{\text{T}}_{48} & \text{IKL}{\text{T}}_{56} & \text{IKL}{\text{T}}_{64} \end{array}\right]\\ ={\left[\begin{array}{cccccccc} v_{1} & v_{9} & v_{17} & v_{25} & v_{33} & v_{41} & v_{49} & v_{57}\\ v_{2} & v_{10} & v_{18} & v_{26} & v_{34} & v_{42} & v_{50} & v_{58}\\ v_{3} & v_{11} & v_{19} & v_{27} & v_{35} & v_{43} & v_{51} & v_{59}\\ v_{4} & v_{12} & v_{20} & v_{28} & v_{36} & v_{44} & v_{52} & v_{60}\\ v_{5} & v_{13} & v_{21} & v_{29} & v_{37} & v_{45} & v_{53} & v_{61}\\ v_{6} & v_{14} & v_{22} & v_{30} & v_{38} & v_{46} & v_{54} & v_{62}\\ v_{7} & v_{15} & v_{23} & v_{31} & v_{39} & v_{47} & v_{55} & v_{63}\\ v_{8} & v_{16} & v_{24} & v_{32} & v_{40} & v_{48} & v_{56} & v_{64} \end{array}\right]}^{T}\times \left[\begin{array}{cccccccc} \text{KL}{\text{T}}_{1} & \text{KL}{\text{T}}_{9} & \text{KL}{\text{T}}_{17} & \text{KL}{\text{T}}_{25} & \text{KL}{\text{T}}_{33} & \text{KL}{\text{T}}_{41} & \text{KL}{\text{T}}_{49} & \text{KL}{\text{T}}_{57}\\ \text{KL}{\text{T}}_{2} & \text{KL}{\text{T}}_{10} & \text{KL}{\text{T}}_{18} & \text{KL}{\text{T}}_{26} & \text{KL}{\text{T}}_{34} & \text{KL}{\text{T}}_{42} & \text{KL}{\text{T}}_{50} & \text{KL}{\text{T}}_{58}\\ \text{KL}{\text{T}}_{3} & \text{KL}{\text{T}}_{11} & \text{KL}{\text{T}}_{19} & \text{KL}{\text{T}}_{27} & \text{KL}{\text{T}}_{35} & \text{KL}{\text{T}}_{43} & \text{KL}{\text{T}}_{51} & \text{KL}{\text{T}}_{59}\\ \text{KL}{\text{T}}_{4} & \text{KL}{\text{T}}_{12} & \text{KL}{\text{T}}_{20} & \text{KL}{\text{T}}_{28} & \text{KL}{\text{T}}_{36} & \text{KL}{\text{T}}_{44} & \text{KL}{\text{T}}_{52} & \text{KL}{\text{T}}_{60}\\ \text{KL}{\text{T}}_{5} & \text{KL}{\text{T}}_{13} & \text{KL}{\text{T}}_{21} & \text{KL}{\text{T}}_{29} & \text{KL}{\text{T}}_{37} & \text{KL}{\text{T}}_{45} & \text{KL}{\text{T}}_{53} & \text{KL}{\text{T}}_{61}\\ \text{KL}{\text{T}}_{6} & \text{KL}{\text{T}}_{14} & \text{KL}{\text{T}}_{22} & \text{KL}{\text{T}}_{30} & \text{KL}{\text{T}}_{38} & \text{KL}{\text{T}}_{46} & \text{KL}{\text{T}}_{54} & \text{KL}{\text{T}}_{62}\\ \text{KL}{\text{T}}_{7} & \text{KL}{\text{T}}_{15} & \text{KL}{\text{T}}_{23} & \text{KL}{\text{T}}_{31} & \text{KL}{\text{T}}_{39} & \text{KL}{\text{T}}_{47} & \text{KL}{\text{T}}_{55} & \text{KL}{\text{T}}_{63}\\ \text{KL}{\text{T}}_{8} & \text{KL}{\text{T}}_{16} & \text{KL}{\text{T}}_{24} & \text{KL}{\text{T}}_{32} & \text{KL}{\text{T}}_{40} & \text{KL}{\text{T}}_{48} & \text{KL}{\text{T}}_{56} & \text{KL}{\text{T}}_{64} \end{array}\right]. \end{array}$$

After performing the matrix multiplication for *V* ^*T*^ × KLT = *V* ^*T*^ × *V* × *I*, the 64 pixels IKLT_1_, IKLT_2_,…, IKLT_63_, and IKLT_64_ are given by

(8) IKLT1=v1×KLT1+v2×KLT2+v3×KLT3+v4×KLT4+v5×KLT5+v6×KLT6+v7×KLT7+v8×KLT8,IKLT2=v9×KLT1+v10×KLT2+v11×KLT3+v12×KLT4+v13×KLT5+v14×KLT6+v15×KLT7+v16×KLT8,⋮IKLT63=v49×KLT57+v50×KLT58+v51×KLT59+v52×KLT60+v53×KLT61+v54×KLT62+v55×KLT63+v56×KLT64,IKLT64=v57×KLT57+v58×KLT58+v59×KLT59+v60×KLT60+v61×KLT61+v62×KLT62+v63×KLT63+v64×KLT64.

If we modify the eight coefficients in *V* ({*v*_*i*_∖1 ≤ *i* ≤ 8}), the distortions will be spread on the totality of the KLT ({KLT_*i*_∖1 ≤ *i* ≤ 64}) matrix. This means that the underlying image will be modified. When the IKLT matrix is computed, significant modifications in the components of the first row are produced. This feature enables us to easily identify the exact location of the watermark. Therefore, it can be concluded that a change in *V* ({*V*_*i*_∖1 ≤ *i* ≤ 8}) produces a noticeable (and thus detectable) change in the first column of the IKLT matrix.

### 6.2. Embedding Steps

Our approach is based on the combination of Haar Wavelet and Karhunen Loeve Transforms for hiding the watermark. The embedding process is described as follows.

- By using the Haar Wavelet Transform, we decompose the original image into second level subband. After that, LH2, HL2, and HH2 are extracted and decomposed into subblocks sized 8 × 8.
- The Karhunen Loeve Transform is applied on the different subblocks.
- Using a secret key, we generate a pseudorandom sequence Key (key_*i*_∖1 ≤ *i* ≤ 8) in which each number can take a value of either 0 or 1. Then this sequence is multiplied by the visibility factor, related to the subblock to be watermarked.
- With the *n*th block to be watermarked, the *n*th value of the index table, and the binary sequence generated by the key (after multiplication by the final value of *α*), the embedding process can be initiated. Depending on the value “*K*” ({*K*∖(0  to  7}) of the index table, the values of the (*K*th + 1) column of eigenvector *V* ({*V*_*i*_∖(1  to  8}) are combined with Key multiplied by *α*. This step will be stopped after we finish the *P* values of different indexing tables.
- Perform the Inverse Karhunen Loeve Transform.
- Perform the Inverse Haar Wavelet Transform to obtain the watermarked image.
- Display watermarked image.

**Figure 9:**
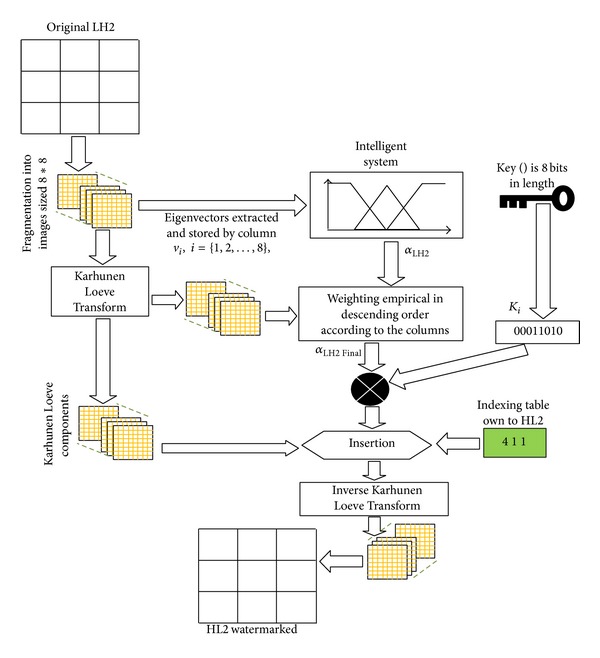
Insertion algorithm applied on LH2.

Figure 9 shows the insertion algorithm applied on the second subband LH2 (the same steps are applied on the HL2 and HH2).

**Figure 10:**
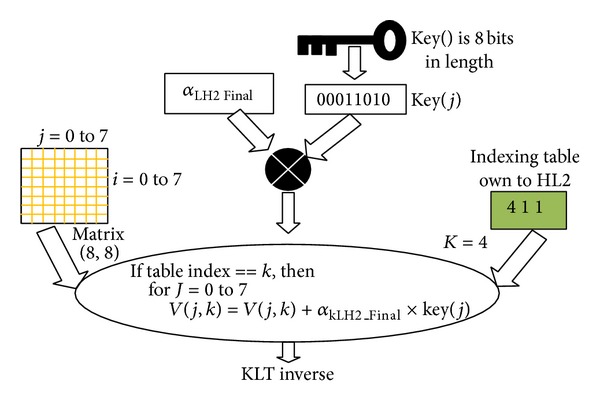
Insertion step in the matrix 8 × 8 applied on the Karhunen Loeve components.

Figure 10 shows the insertion step in the matrix 8 × 8 on the Karhunen Loeve component.

## 7. Proposed Extraction Algorithm

Generally, the extraction phase follows the reverse steps with respect to the insertion. We have the original image “*I*” and the watermarked image “*I*_*w*_.”

(i) The second Haar Wavelet Transform is applied on the original and watermarked images.
(ii) Extract the subbands of the second decomposition LH2, HL2, and HH2.
(iii) Decompose the different subbands into subblocks sized 8 × 8.
(iv) Compute the difference, all the blocks (two by two) between subblocks own to I and those related to *I*_*w*_.
(v) In the proposed method, the inserted watermark is the binary sequence generated by a secret key after their multiplying by the visibility factor. For extraction, the resulting difference 8 × 8 matrices (*M*_*D*_) are compared, term by term, with threshold noted *T* (*T* is determined empirically):
  (9) if    MD(i,j)≥T⟹MD(i,j)=1else    MD(i,j)≺T⟹MD(i,j)=0,{i,j∖1  to  8}.
(vi) Calculate the correlation between the secret key and all row of *M*_*D*_. The number of the column containing the maximum correlation value indicates the extracted index value ({index  value∖0 ≤ *i* ≤ 7}).

**Figure 11:**
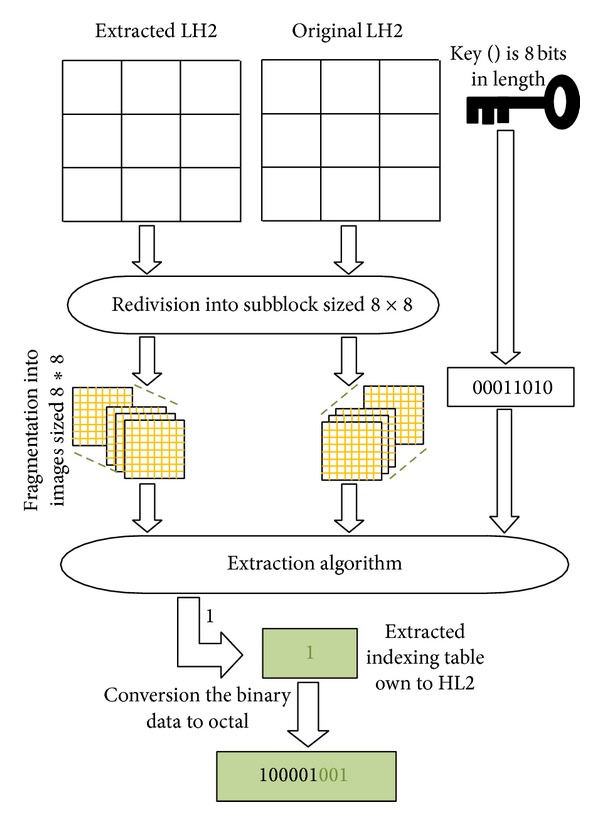
Extraction Step.

Figure 11 illustrates the different steps to follow for extracting the index table related to LH2 (the same step is applied on the watermarked subbands HL2 and HH2).

(vii) Concatenate all indexing tables extracted from different subbands, and convert them to binary data.
(viii) Apply the decoder algorithm (serial Turbocode) [22].

Finally, the patient data is extracted, verified, and eventually corrected.

## 8. Validation of the Proposed Method

For medical images, the watermark must be imperceptible. The watermarked image should be widely similar to the original image so as not leading to a misdiagnosis.

The validity of any watermarking algorithm can become more important than testing it against various attacks types.

For this, we subject the watermarked medical image to a series of attacks and test the sensitivity of the watermark and its ability to detect any change in the image. After application of each attack, the entirety of embedded watermark is extracted and compared through similarity analysis with the original marks (*W*_original_, *W*_extracted_) to ensure that these marks are not damaged by the attacks applied on the image.

### 8.1. Watermark Detection Tools

The measure of “degree of reliability” of the detected watermark is accomplished by the “calculation of distances” between the inserted and detected watermark. This measure is carried out using the Normalized Cross-Correlation [13]. The Normalized Cross-Correlation (NC) of two signals consists in computing their dependence. The NC is defined as

(10) $$\begin{array}{c} \text{NC}\left(W,\bar{W}\right)=\frac{\sum_{i}\sum_{j}\left(W_{ij}-w_{w}\right)\left({\bar{W}}_{ij}-w_{\bar{w}}\right)}{\sqrt{\sum_{i}\sum_{j}{\left(W_{ij}-w_{w}\right)}^{2}}\sqrt{\sum_{i}\sum_{j}{\left({\bar{W}}_{ij}-w_{\bar{w}}\right)}^{2}}}, \end{array}$$

where *W*, $\overset{-}{W}$ indicate, respectively, the original and the extracted watermark and *w*_*w*_ and $w_{\overset{-}{w}}$ correspond, respectively, to the mean of the original and extracted watermark. In the literature, a NC value which is equal or above 0.75 denotes an acceptable extracted watermark [23].

### 8.2. Peak Signal to Noise Ratio (PSNR)

Among the most important distorting measures in image processing is the Peak Signal to Noise Ratio, PSNR [25]. It is an assessment of the decibel difference between the original image and one that is processed. In fact, a PSNR below 30 dB image can be considered useless. The PSNR is defined by the following formula:

(11) (PSNR)dB =10 log10{N×M[max⁡⁡I2(i,j)∑i,j[I(i,j)−Iw(i,j)]2]},

where *M* and *N* are the number of rows and columns of the image which contains *M* × *N* pixels, *I* is the host image, and *I*_*w*_ is the watermarked image.

### 8.3. Weighted Peak Signal to Noise Ratio (WPSNR)

The Peak Signal to Noise Ratio PSNR is based on comparing pixel to pixel the original image and the received watermarking image. The WPSNR proposed by Voloshy Noviskiand and al. [26] is defined by the following formula:

(12) (WPSNR)dB =10 log10{M×Nmax⁡I2(i,j)∑i,j[(I(i,j)−Iw(i,j))/(1+varI(i,j))]2}.

With var(*i*, *j*) representing the local variance of pixel at location (*i*, *j*), *I*(*i*, *j*) is the intensity value of the pixel (*i*, *j*) in the original image, and *I*_*w*_(*i*, *j*) the intensity value of the pixel in the image under test. *M* and *N* are, respectively, the height and width of the image.

### 8.4. Experimental Results

Regardless of the domain or the method for hiding of the watermark, it is very important to have a good PSNR and WPSNR values; this is especially true in the medical imaging domain. This work has been applied to different Radiographic images sized of 512 × 512 pixels of resolution of 8 bits/pixel. We start analysing our experimental results by a preliminary study, that is to say, a study without the application of attacks on the watermarked images.

**Figure 12:**
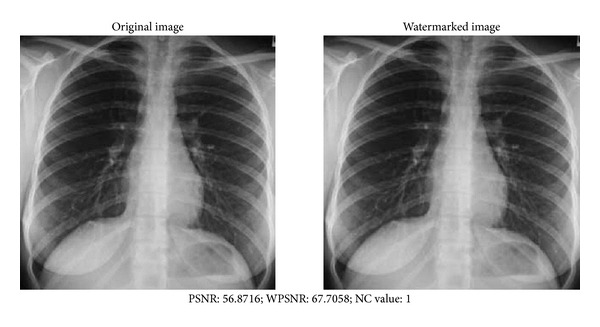
Original and watermarked Radiographic images.

Figure 12 shows the original medical images and watermarked ones. We notice that the Human Visual System does not distinguish the difference caused by the marking.

The first test of robustness for an attack is the application of the JPEG 2000 compression. It must be noted that the image compression algorithms are particularly aggressive for watermarked images. We have chosen to apply different image compression ratios to the watermarked images as shown in Table 1 which presents the results of simulations showing the NC values between the original and extracted watermark after image compression attacks.

After applying the JPEG2000 image compression attacks, we remark that even when varying the compression rate factor between 10% and 70%, the NC factor remains equal to 1. We conclude that the proposed approach makes the inserted watermarks resistant to this type of attacks.

The second kind of attacks tests is the application of several types of digital filters. In our experiments, we applied median, wiener, and low pass digital filtering attacks.  Table 2 shows PSNR, WPSNR, and the Normalized Cross-Correlation (NC) factor of Radiographic watermarked image robustness of our watermarking schema against these attacks with different window sizes of the filters.

We notice that our proposed method is very effective against these types of attacks (NC is equal to 1 regardless of the size of the filter).

The third attack is the application of two types of noise: Gaussian noise and Salt & Pepper noise. Tables 3 and 4 illustrate the PSNR, WPSNR, and the NC before watermarking and after applying these attacks.

In general, it is necessary to test our schema against noise. The applied attacks are Gaussian noise (with different variance factors) and Salt & Pepper noise attack (varying the density factors). The different tests show that the proposed method attains good results, with NC = 1 for most of the cases.

The fourth type of attack applied is the geometric transform such as the rotation attack and cropping.  Table 5 shows the PSNR, WPSNR, and the NC values after applying the rotation attack according to the rotation angle.  Table 6 shows the PSNR, WPSNR, and the NC values after applying the cropping attack with various sized window.

Among the most dangerous attacks applied on a several watermarking algorithms are the geometric transforms. The proposed tests are the cropping attack (with different windows) and the rotation attack. The obtained results give us an NC equal or close to 1.

### 8.5. Evaluation of the Proposed Algorithm

In this section, the proposed watermarking method is investigated by comparing our results to those cited in the subsection of the related work.

Comparing the psychovisual quality of the original image and the watermarked image, the proposed algorithm yields very good results. In the absence of attacks, the PSNR equal to 56.8716 and WPSNR equal to 67.7058 are obtained, yielding results that are approximately equal or often better than those algorithms previously cited in the related section of the works.

After applying many attacks, it is necessary to evaluate the Normalized Cross-Correlation (NC). Among the most serious attacks is the attack by image compression such as JPEG 2000. In present paper, our applied algorithm is very effective against this kind of attacks. The NC value stills equal to 1 when the rate of image compression goes from 10% to 70%. This obtained result is more accurate than all results quoted precisely where the NC decreases with the increase of the image compression rate. To test our method against attacks based on digital filtering, many filter types are applied such as median filter, low-pass filter, and wiener filter, with various sized windows ([3 × 3], [5 × 5], [7 × 7], [9 × 9]). The obtained results of the NC are equal to 1.

To evaluate the proposed method against the noise, we attacked watermarked images by two types of noises such as the Salt & Pepper and Gaussian noise. We obtained very promising results; the NC is always equal to 1.

We also tested our method against geometric transforms attacks (cropping and rotation). The obtained NC values are very close to 1. Compared to the previous works, our proposed method gives results near to those found in the literature.

## 9. Conclusion

The present work is a new robust watermarking algorithm combining the Haar Wavelet and the Karhunen Loeve Transforms. The main contribution of this paper consists in improving the three principal factors existing in all watermarking systems (robustness, imperceptibility, and integration rate). To do so, we came across many steps.

In order to improve the factor of imperceptibility, we used the high frequency (second subband of the Haar Wavelet Transform) to hide the watermark. The Fuzzy Inference System is used to determine the visibility factor according to the proper characteristics of the insertion plan. Each subband (LH2, HL2, and HH2) is subdivided into subblocks sized 8 × 8. Then, the Karhunen Loeve Transform is applied in order to decorrelate the different wavelets coefficients. Indexing tables are then used to choose the location of components supporting the watermark. At this stage, the visibility factors determined by the FIS (*α*_*LH*⁡2_, *α*_HL2_, and *α*_HH2_) are adapted according to the weights of the eigenvalues to determine the final visibility factors (*α*_*LH*⁡2_Final_, *α*_HL2_Final_, and *α*_HH2_Final_).

Our contribution concerning the robustness is the use of the ECC by means of the serial Turbocode. We obtained good results in terms of the extracted watermark which is similar to the original. Our principal idea to increase the integration rate by a factor 3 consists in inserting the octal representation of the watermark.

To evaluate the performance of our method, the proposed system is applied on medical images. Several tests are performed, such as digital filtering, JPEG 2000 compression, adding noise, and geometric transformation. The results show that our method is very robust against these attacks. It supports image compression attacks such as JPEG 2000 up to 70% compression ratio.

Our experiments have also shown that our method is resistant to digital filtering attacks. We noticed that the extracted watermark is similar to the original watermark.

To evaluate the resistance of our proposal against the geometric transformation attacks, image rotation techniques and cropping are applied on watermarked images. Here again, the watermark extraction was faithful.

## Figures and Tables

**Algorithm 1:**
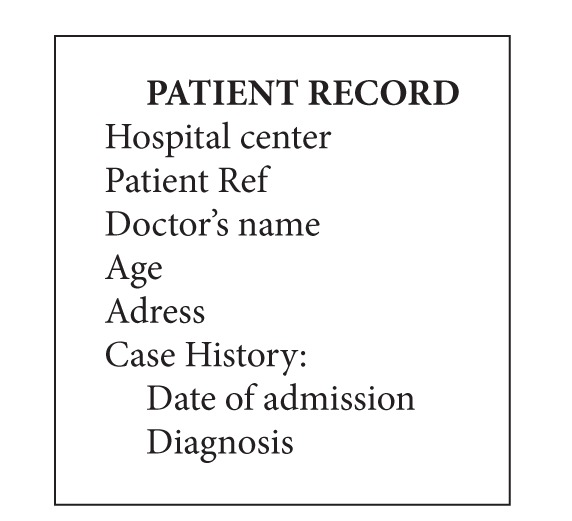
Example of the data patient to be inserted.

**Table 1:** Evaluation of the PSNR, WPSNR, and the Normalized Cross-Correlation values of the watermarked and attacked images by a JPEG 2000 image compression Algorithm.

Rate (%)	PSNR (dB)	WPSNR (dB)	NC
10	55.7797	44.9018	**1.0000**
20	52.5173	42.1029	**1.0000**
30	52.0727	42.1458	**1.0000**
40	50.9338	41.2982	**1.0000**
50	49.2004	39.8061	**1.0000**
60	48.6195	39.3886	**1.0000**
70	47.3179	38.5497	**1.0000**
80	45.0417	37.0304	**0.7358**
90	40.8006	33.7765	**0.5422**

**Table 2:** Evaluation of the algorithm against filters attacks.

Filter's window size	Median filter			Low pass filter			Wiener filter		
	PSNR (dB)	WPSNR (dB)	NC	PSNR (dB)	WPSNR (dB)	NC	PSNR (dB)	WPSNR (dB)	NC
[3 × 3]	45.9121	57.1649	**1.0000**	35.5290	46.4338	**1.0000**	35.1327	49.0685	**1.0000**
[5 × 5]	41.5136	50.0277	**1.0000**	32.5677	40.7808	**1.0000**	31.5552	41.9205	**1.0000**
[7 × 7]	38.3304	45.4194	**1.0000**	30.6348	37.6424	**1.0000**	29.4059	37.8203	**1.0000**
[9 × 9]	36.1094	42.5076	**1.0000**	29.2368	35.6657	**1.0000**	27.8707	35.2772	**1.0000**

**Table 3:** PSNR, WPSNR, and Normalized Cross-Correlation for watermarked and attacked by Salt & Pepper noises.

Density	PSNR (dB)	WPSNR (dB)	NC
0.01	33.1542	46.4909	**1.0000**
0.02	30.9053	44.8789	**1.0000**
0.03	29.5090	43.8205	**1.0000**
0.04	28.7254	43.1368	**1.0000**
0.05	27.6397	42.1583	**1.0000**
0.06	26.9567	41.5865	**1.0000**
0.07	26.4402	41.0837	**1.0000**
0.08	25.9215	40.6158	**0.9938**
0.09	25.4346	40.1982	**1.0000**

**Table 4:** PSNR, WPSNR, and Normalized Cross-Correlation for watermarked and attacked by Gaussian noise.

Variance	PSNR (dB)	WPSNR (dB)	NC
0.001	44.3893	31.2215	**1.0000**
0.002	42.7730	28.9659	**1.0000**
0.003	41.8008	27.4845	**1.0000**
0.004	40.9993	26.3917	**1.0000**
0.005	40.2247	25.4987	**1.0000**
0.006	39.6044	24.7603	**1.0000**
0.007	39.2108	24.1527	**1.0000**
0.008	38.7113	23.6068	**1.0000**
0.009	38.3282	23.1109	**0.9959**

**Table 5:** PSNR, WPSNR, and Normalized Cross-Correlation for watermarked and attacked by image rotation transform.

Rotation angle (°)	PSNR (dB)	WPSNR (dB)	NC
1	26.7254	34.2455	**1.0000**
5	19.7487	25.8241	**1.0000**
11	16.8277	22.8616	**1.0000**
15	15.7812	21.8145	**0.9728**
19	15.0400	21.0650	**0.9812**
23	14.4720	20.4955	**0.9854**
27	14.0551	20.0784	**0.9621**
31	13.7572	19.7826	**0.9474**
35	13.5653	19.5880	**0.9455**
39	13.4471	19.4679	**0.9285**
45	13.4550	19.4698	**0.9645**

**Table 6:** PSNR, WPSNR, and Normalized Cross-Correlation for watermarked and attacked by “cropping.”

Window size	PSNR (dB)	WPSNR (dB)	NC
[32 × 32]	35.0841	41.1315	**1.0000**
[64 × 64]	24.2723	30.2761	**1.0000**
[96 × 96]	19.9352	25.9459	**1.0000**
[128 × 128]	17.5275	23.5390	**0.9896**
[160 × 160]	16.1426	22.1554	**0.9793**
